# Comparative Metagenomic Analysis of Coral Microbial Communities Using a Reference-Independent Approach

**DOI:** 10.1371/journal.pone.0111626

**Published:** 2014-11-07

**Authors:** Camila Carlos, Daniel Bedo Assumpção Castro, Laura M. M. Ottoboni

**Affiliations:** Center for Molecular Biology and Genetic Engineering (CBMEG), State University of Campinas – UNICAMP, Campinas, São Paulo, Brazil; Graz University of Technology (TU Graz), Austria

## Abstract

By comparing the SEED and Pfam functional profiles of metagenomes of two Brazilian coral species with 29 datasets that are publicly available, we were able to identify some functions, such as protein secretion systems, that are overrepresented in the metagenomes of corals and may play a role in the establishment and maintenance of bacteria-coral associations. However, only a small percentage of the reads of these metagenomes could be annotated by these reference databases, which may lead to a strong bias in the comparative studies. For this reason, we have searched for identical sequences (99% of nucleotide identity) among these metagenomes in order to perform a reference-independent comparative analysis, and we were able to identify groups of microbial communities that may be under similar selective pressures. The identification of sequences shared among the metagenomes was found to be even better for the identification of groups of communities with similar niche requirements than the traditional analysis of functional profiles. This approach is not only helpful for the investigation of similarities between microbial communities with high proportion of unknown reads, but also enables an indirect overview of gene exchange between communities.

## Introduction

Metagenomics revolutionized microbial ecology and environmental microbiology because it allows the study of genes and functions of the whole microbial community of an environment, including uncultured micro-organisms. The number of metagenomic studies is growing fast in the last few years, especially due to the rising of ultra-high throughput sequencing technologies. Comparing two or more metagenomics datasets derived from different environments is one common approach used to identify functions and genes that play an important role in one of these environments. Usually, these comparisons are made through functional profiles derived from the annotated sequences according to a reference database, such as SEED, Pfam and COGs. Nevertheless, in several cases, most of the metagenomic sequences cannot be allocated to one of these functional categories due to dissimilarity between newly generated and reference database sequences [1]. Thus, ignoring the portion of unknown sequences during comparative metagenomics can lead to biased conclusions. Some authors have developed comparative analyses of metagenomes using a reference-independent approach. Dutilh et al. [1] proposed the use of cross-assembling to identify similarity relationships among metagenomes. Using cross-assembling files from two different datasets, the crAss method enables the calculation of distances between the metagenomic sample pairs using the number of reads from each metagenome that was used to build a contig. Despite the high efficiency of this method of metagenome comparison, it is computationally intense and time-consuming, especially for requiring assembling of sequences, which is particularly difficult in the case of complex sequence datasets and requires several gigabytes of available RAM memory [2]. Therefore, in this study, a database-independent approach was used to compare different metagenomes (two coral metagenomes sequenced in this work and 29 metagenomes publicly available at MG-RAST) by all-against-all BLAT of the reads (with a threshold of 99% of nucleotide identity and minimum alignment length of 100 bp), in order to find nearly identical sequences that were shared between metagenome pairs. Our hypothesis was that similar microbial communities, with similar functional composition or that harbour the same biological entities (bacterial strains or plasmids, for example) share, together, more identical sequences than distinct communities. The two coral species (*Mussismilia hispida* and *Madracis decactis*) utilized in this study are the only scleractinian corals from the São Paulo State coast, and the study of *M. hispida* is of particular interest because it belongs to a genus endemic to the Brazilian coast.

An advantage of this approach is that the presence of shared identical sequences may also be indicative of recent events involving gene swapping between environments. Using a similar approach, Kloesges et al. [3] analyzed 329 proteobacteria genomes and found that most of the gene sharing was among bacteria from different taxa inhabiting the same habitat. Smillie et al. [4] assessed recently transferred genes (more than 99% nucleotide identity) among 2,235 bacterial genomes, and also found that the habitat influenced these events, rather than geographical distance or taxonomy. Although there have been several comprehensive studies of gene transference among bacterial genomes, few investigations have considered the way in which genes can move between different environments. Forsberg et al. [5] recently reported multi-drug-resistant soil bacteria containing resistance genes with identical nucleotide sequences to genes from human pathogens, indicative of recent gene exchange events between environmental bacteria and clinical pathogens. However, the authors did not suggest ways in which these genes could be transferred. Thus, the indirect study of the frequency and dynamics of gene swapping events among environments, provided by the approach proposed in this work, may help to understand how genes, such as the ones related to antibiotic resistance, are spread over large-scale geographic distances.

## Materials and Methods

### Sample collection

One piece of colony (around 5 cm^2^) of each of the corals *Mussimilia hispida* and *Madracis decactis* were collected in March 2012 at Buzios Island, São Paulo State, Brazil (23° 48' 157″ S, 45° 07' 181″ W). The sea surface temperature was 22°C, and the coral samples were collected at a depth of 11 m. The colonies were transported to the land in a sterile box with seawater, then briefly rinsed with autoclaved seawater and stored at −20°C for two days prior to DNA isolation.

The specimens were sampled and provided by CEBIMar-USP (Centro de Biologia Marinha da Universidade de São Paulo). The sampling location is a public beach, thus no specific permissions were required to collect the material necessary for the present study.

### DNA isolation and sequencing

The coral pieces were vigorously washed with TE buffer (10 mM Tris, 1 mM EDTA, pH 8.0) in a vortex mixer. The solution obtained was centrifuged for 30 s at 8944 RCF units (g) to pelletize the debris. The supernatant was transferred to another tube and centrifuged for 10 min at 15115 g. The pellet was employed for DNA isolation using a Wizard Genomic DNA Purification Kit (Promega, Madison, Wiscosin, USA), according to the manufacturer's instructions for Gram-positive bacteria. Average yield was 2 ng/µL of DNA for each sample. The DNA was amplified using a REPLI-g Midi-kit (QIAGEN, Duesseldorf, Germany), according to the manufacturer's instructions. The DNA was quantified using a Qubit Kit (Invitrogen, Carlsbad, California, USA), and the integrity was confirmed by 1% agarose gel electrophoresis. The DNA obtained ranged between 48,000 and 12,000 bp, and was sequenced by using 454 technology on a Roche GS FLX platform at the DNA Facility of Iowa State University.

### Sequence and statistical analysis

Reads were trimmed by quality (minimum of 25) and length (minimum of 100 bp) using PRINSEQ [6]. The barcodes were removed with TagCleaner software [7]. Reads showing 95% of nucleotide identity with sequences of vectors or mitochondrial databases (ftp://ftp.ncbi.nih.gov/refseq/) were removed by using the script exclude_seqs_by_blast.py. The sequences were de-replicated and annotated in SEED subsystems (e-value <1e-05) using the automatic MG-RAST platform [8], and the metagenomes of *M. hispida* and *M. decactis* were deposited under the accession numbers 4516694.3 and 4516541.3, respectively. In addition, the de-replicated reads were also submitted to CoMet [9] to identify the Pfam domain families (e-value <0.001) (http://pfam.sanger.ac.uk/). The reads of the metagenomes were also submitted to MetaVir, a web-server for virome analysis [10].

One-way analysis of similarity (ANOSIM) and non-metric multidimensional scaling (MDS), using Bray-Curtis dissimilarity, were performed with PAST software (http://folk.uio.no/ohammer/past/). The differentially abundant functions and Pfam domains were identified using White's non-parametric t-test with 1,000 bootstrapping replications [11]. The differentially abundant SEED functions where identified with STAMP software (http://kiwi.cs.dal.ca/Software/STAMP). Comparisons between *M. hispida* and *M. decactis* libraries were performed using bootstrapping and Bonferroni correction of p-values; Comparison between the coral metagenomes sequenced in this work and the 29 metagenomes publicly available on MG-RAST were also performed in STAMP, using White's non-parametric t-test and Bonferroni correction of p-values.

In order to detect identical sequences shared among environments, an all-against-all blat (minimum identity 99% and minimum alignment length  = 100 bp) was performed with the de-replicated sequences of the metagenomes. A matrix of pairwise Jaccard's similarities was built using the formula: J_AB_  =  (H_AB_ + H_BA_)/(N_A_ + N_B_), where H_AB_ is the number of hits from metagenome A, using metagenome B as the database, H_BA_ is the number of hits from metagenome B, using metagenome A as the database, and N_A_ and N_B_ are the total numbers of reads of metagenomes A and B, respectively. Mantel's test was used to assess the correlation of the Jaccard's indexes of shared identical sequences with the geographical distance and the Bray-Curtis similarity indexes of taxonomic (at Class level) and functional composition (at SEED level 2, 3, and Pfam domains), using PAST software. A gene-sharing network was visualized using Cytoscape v. 3.0 software, and the metagenomes were represented by the nodes and the edges showing Jaccard's similarity values equal to or greater than 1.0×10^−6^. A network was produced using a prefuse force directed algorithm, weighted with Jaccard's values. Nodes were clustered using the ModuLand plug-in [12], which searches for groups with a high density of internal edges, weighted with the Jaccard's values.

## Results

### Description of the metagenome of the Brazilian corals

**Figure 1:**
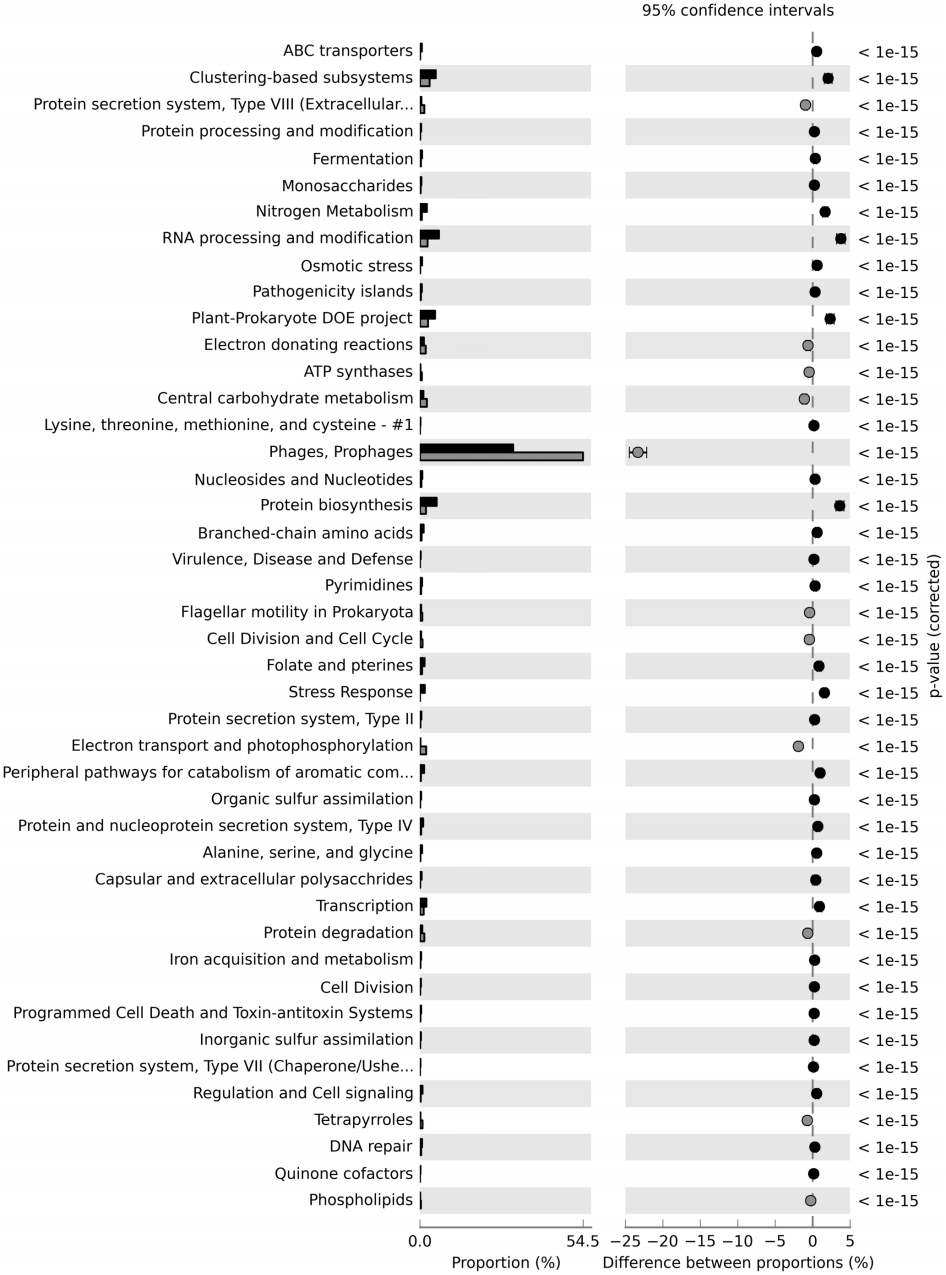
Distribution of the SEED categories (Level 2) with difference between the metagenomes of *M. hispida* (light gray) and *M. decactis* (dark gray).

A total of 368,772 good quality reads were obtained for *Mussimilia hispida*, with an average length of 446 bp, and 293,580 for *Madracis decactis*, with an average length of 453 bp. Only 4.4% (16,910) of the reads from *M. hispida* and 3.3% (9,688) of the reads from *M. decactis* could be annotated by the hierarchical SEED subsystem, and 8.2% (30,239) of the reads from *M. hispida* and also 8.2% (24,073) of *M. decactis* presented Pfam hits. Table 1 shows the top ten Pfam domains and SEED functions identified for each coral species; a high abundance of phage and virus proteins can be seen for both classification systems and libraries. The features (at Level 2) that were different for the two metagenomes and presented the lowest p-values and the highest effect size are shown in Figure 1. For example, “ABC transporters” and “Clustering-based subsystems” were enriched in the metagenome of *M. decactis*, and “Protein secretion system, type VIII” was enriched in *M. hispida*.

**Table 1:** Functional classification of *M. hispida* and *M. decactis* reads performed using MG-RAST and CoMet platforms.

*Mussismilia hispida*			*Madracis decactis*		
Pfam	Pfam Description	% of total reads	Pfam	Pfam Description	% of total reads
PF02305	Capsid protein (F protein)	1.8659	PF02407	Putative viral replication protein	1.6346
PF00910	RNA helicase	0.8976	PF00910	RNA helicase	1.1833
PF02407	Putative viral replication protein	0.6847	PF01446	Replication protein	0.7610
PF00124	Photosynthetic reaction centre protein	0.5396	PF06280	Fn3-like domain (DUF1034)	0.5338
PF01844	HNH endonuclease	0.2386	PF01844	HNH endonuclease	0.4881
PF01446	Replication protein	0.2251	PF05127	Putative ATPase (DUF699)	0.4190
PF09295	ChAPs (Chs5p-Arf1p-binding proteins)	0.1098	PF08019	Domain of unknown function (DUF1705)	0.3546
PF01051	Initiator replication protein	0.1096	PF02305	Capsid protein (F protein)	0.3406
PF06280	Fn3-like domain (DUF1034)	0.1003	PF04127	DNA/pantothenate metabolism flavoprotein	0.2742
PF00006	ATP synthase alpha/beta family	0.0998	PF00799	Geminivirus Rep catalytic domain	0.2153
SEED Function		% of total reads	SEED Function		% of total reads
Phage protein		0.01247	Phage protein		0.0361
Gamma-carotene hydroxylase		0.00597	Ribonucleotide reductase of class Ia (aerobic), alpha subunit (EC 1.17.4.1)		0.0061
Heat shock protein 60 family chaperone GroEL		0.00569	Gamma-carotene hydroxylase		0.0061
COG0009 Sua5 subfamily, required for N6-threonylcarbamoyl adenosine t(6)A37 modification in tRNA		0.00569	Heat shock protein 60 family chaperone GroEL		0.0051
Ribonucleotide reductase of class Ia (aerobic), alpha subunit (EC 1.17.4.1)		0.00542	COG0009 Sua5 subfamily, required for N6-threonylcarbamoyl adenosine t(6)A37 modification in tRNA		0.0051
ATP synthase beta chain (EC 3.6.3.14)		0.00515	GTP-binding protein		0.0044
CoB—CoM heterodisulfide reductase subunit C (EC 1.8.98.1)		0.00515	Nitrite reductase probable electron transfer 4Fe-S subunit (EC 1.7.1.4)		0.0044
Photosystem II protein D2 (PsbD)		0.00461	Decarboxylase		0.0041
Xaa-Pro aminopeptidase (EC 3.4.11.9)		0.00380	Translation elongation factor LepA		0.0031
Type I restriction-modification system, specificity subunit S (EC 3.1.21.3)		0.00380	GMP synthase [glutamine-hydrolyzing] (EC 6.3.5.2)		0.0027

Based on MG-RAST taxonomic classification, most of the annotated reads of *M. hispida* and *M. decactis* were classified as Bacteria. In both datasets, most of the bacterial reads were classified as Proteobacteria and the most abundant order was Pseudomonadales, corresponding to 25.1% (4,290) of the bacterial reads in *M. hispida* and 21.6% (4,052) of the bacterial reads in *M. decactis*. Conversely, according to CoMet Pfam classification, most of the reads, for both corals, were classified as VMG (Viral Metagenome) (Table 2). According to the MetaVir taxonomic classification, 30,619 (∼10.4%) reads of the *M. decactis* metagenome were of virus origin, and 78% (23,872) of these reads were classified as ssDNA viruses, with the Circoviridae family being the most representative, with 31% of the viral reads (9,392). In the metagenome of *M. hispida*, 34,160 (9.2%) reads were from viruses, with 80% (27,191) classified as ssDNA viruses and the most representative family being the Microviridae/Gokushovirinae, with 29% (9,829) of the viral reads.

**Table 2:** Taxonomic classification of *M. hispida* and *M. decactis* reads performed using MG-RAST and CoMet platforms.

	MG-RAST Taxonomic classification (%)				CoMet (TaxyPro) Taxonomic classification (%)				
	Archaea	Bacteria	Eukaryota	Viruses	Archaea	Bacteria	Eukaryota	VMG	Viruses
*Mussismilia hispida*	1.39	57.76	29.31	1.94	0.43	12.00	19.2	62.4	5.9
*Madracis decactis*	0.89	65.26	15.30	5.77	0.22	11.5	7.1	72.4	8.3

VMG  =  viral metagenomes.

### Comparative metagenomics

The Pfam and SEED function distributions of the *M. hispida* and *M. decactis* metagenomes were compared with 29 metagenomes publicly available at MG-RAST, including the metagenomes of two coral species, human gut, chicken cecum, invertebrates, seawater, soil, mines, plasmidomes of cow rumen and activated sludge, and viromes of cystic fibrosis lung and seawater (Table S1).

The distribution of Pfam domains in the coral metagenomes was significantly different from the other metagenomes (one-way ANOSIM, R = 0.8277, p = 0.0069). Those overrepresented in the Brazilian coral metagenomes were: PF02674 (difference around 108-fold, p<0.0001), related to colicin V production in *E. coli*; PF02892, a BED-type zinc finger domain related to eukaryotic transposases (difference around 10-fold, p = 0.0009); PF03239 (difference around 33-fold, p = 0.0002), described as an iron permease FTR1 family; PF07903, described as PaRep2a protein of unknown function (difference around 2-fold, p = 0.0006); PF08668, involved in nucleic acid metabolism and signal transduction (difference around 11-fold, p = 0.0002); PF10111, a glycosyltransferase-like family 2 (difference around 136-fold, p = 0.0005); and PF11654, a domain of unknown function that seems to be involved in protein export (difference around 316-fold, p<0.0001).

**Figure 2:**
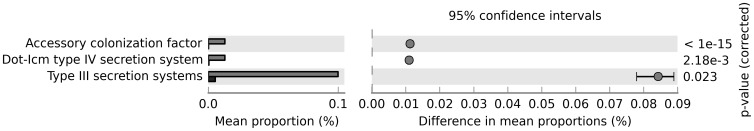
Distribution of the SEED categories (Level 3) with difference between the metagenomes of *M. hispida* and *M. decactis* (light gray) and the 29 metagenomes publicly available at MG-RAST (dark gray).

At SEED Level 2, the differences between the present data and publicly available datasets were significant (one-way ANOSIM, R = 0.5653, p = 0.0328). At SEED Level 3, the metagenomes of *M. hispida* and *M. decactis* were also more similar to each other than to the other metagenomes (one-way ANOSIM, R = 0.5949, p = 0.0285). The functional categories at SEED Level 3 that showed significant differences between the metagenomes of the Brazilian corals and all the other metagenomes were identified using White's non-parametric t-test with 1,000 bootstrapping replications. Figure 2 shows the attributes where the differences presented the smallest p-value. The only SEED categories that were differentially distributed were “Accession colonization factor”, “Dot-Icm type IV secretion system”, and “Type III secretion systems”, which were more abundant in the metagenomes of the Brazilian corals.

The visualization of similarities among the metagenomes by non-metric MDS of SEED functional categories and Pfam profiles did not result in clear discriminations of the communities (Figures S1 and S2).

No Pfam domains or SEED categories were found to be unique to the coral metagenomes used in this study (*M. hispida*, *M. decactis*, *Porites compressa* and *Acropora* sp.).

### Gene-sharing network

An all-against-all approach was used to look for nearly identical sequences (99% of nucleotide identity and minimum alignment length of 100 bp) in pairs of metagenomes, in order to obtain their similarity indexes (Table S2). The metagenomes that shared more identical sequences were *M. decactis* and *M. hispida* (J = 9.152×10^−4^). The human gut metagenome TS5 shared more identical sequences with the chicken cecum sample (J = 3.47×10^−4^) than with the human gut TS1 (J = 1.53×10^−4^). Other metagenomes that shared a high number of identical sequences were the marine sponges SpongeAb1 and SpongeAb2 (J = 4.21×10^−4^), and the artic viromes ArcticVir and GOMVir (J = 3.13×10^−4^). Pfam domains were identified in the shared sequences of the four pairs of metagenomes that most shared sequences (*M. hispida* and *M. decactis*; ArcticVir and GOMVir; SpongeAb1 and SpongeAb2; and Gut_Ts5 and ChickenCecum). The top ten Pfam hits are shown in Table S3. However, on average, Pfam domains were identified in only 26% of the sequences. The Jaccard's values of shared sequences were not strongly correlated with the taxonomic profile at superkingdom level (Mantel's test, R = 0.1983, p = 0.0002), the SEED functional profile at level 3 (Mantel's test, R = 0.1172, p = 0.0028), and the Pfam distribution (Mantel's test, R = 0.1892, p = 0.0002). This means that the metagenome pairs that were more similar in terms of functional and taxonomic (at the superkingdom level) composition did not share more identical sequences. Moreover, no strong correlation was found between sequence-sharing and geographical distance (Mantel's test, R = 0.1638, p = 0.0002), hence a metagenome pair collected at close sample sites did not share more sequences than a metagenome pair collected at distant sample sites. ANOSIM was used to test whether datasets for groups in the same category shared more sequences within the group than between groups. The categories tested were habitat (marine or terrestrial); lifestyle (free-living or animal-associated); climate (tropical, temperate, or polar); metagenome type (microbial, plasmidial, or viral); sequencing methodology (cloning or not); MDA (amplified or not); and study (metagenomes sequenced in the same study were grouped together) (Table 3). The groups that had significant differences in sequence-sharing were marine and terrestrial (ANOSIM, R = 0.2032, p = 0.0003), and tropical and temperate (ANOSIM, R = 0.2109, p = 0.0001).

**Table 3:** One-way analysis of Jaccard's similarities (ANOSIM) using gene-sharing among datasets.

One-way ANOSIM	Habitat	Climate	Lifestyle	Metagenome type	Cloning	MDA	Study
R	0.2032	0.2109	0.1173	0.0558	−0.06622	0.07998	0.1163
p-value	0.0003	0.0001	0.0146	0.1387	0.7968	0.0505	0.2146

A visualization of the similarities among the metagenomes obtained by non-metric MDS of gene-shared hits is presented in Figure S3, for comparison with the functional similarities (Pfam and SEED profiles) using the same technique.

**Figure 3:**
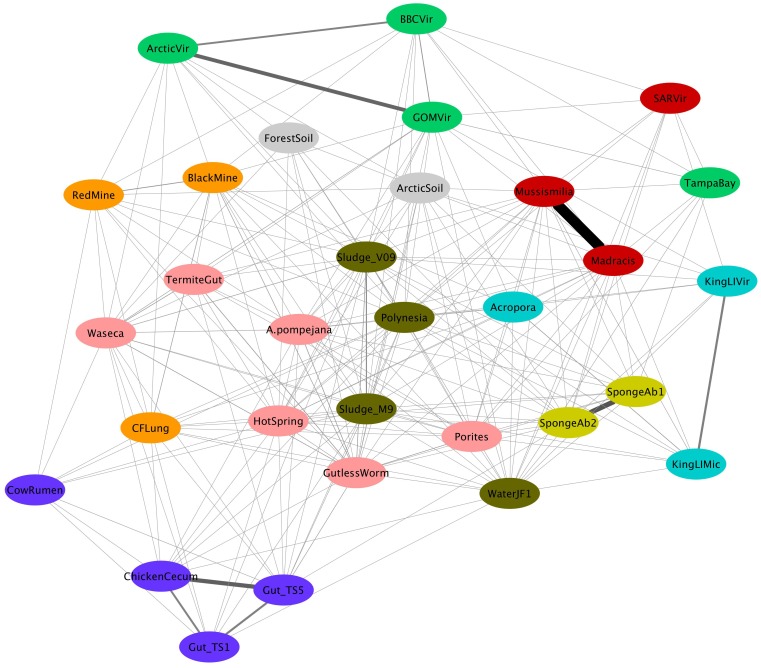
Gene-sharing network among 31 metagenomes obtained using a prefuse force directed algorithm. Metagenomes are represented by nodes. The colours of the nodes represent the module classification of the node. The edges represent Jaccard's connection equal to or greater than 0.000001. The edge thickness is proportional to the Jaccard's value.

Figure 3 shows the gene-sharing network among metagenomes, where thicker edges represent greater Jaccard's values between two metagenomes (nodes). Table S4 shows the values of centrality measures of each node. Polynesia metagenome presented the highest values for degree and betweenness centrality, followed by Sludge_V09 and WaterJF1 (Table S4).

Nine clusters were detected among the metagenomes analyzed. A cluster is a highly dense region in a network, which means that clusters are groups of nodes that share more identical nucleotide sequences. Nodes belonging to the same module are presented in the same colour in Figure 3. For example, *M. hispida*, *M. decatis*, and SARVir belong to the same module, which means that they shared more sequences with each other than with other nodes.

## Discussion

Although very similar, the functional and taxonomic profiles of *M. hispida* and *M. decactis* presented some unique features. Notable were the differences in the dominant viral family. The Circoviridae was the most abundant in the *M. decactis* dataset. Members of this family are usually described as infecting animal cells [13]. The Microviridae family, most abundant in *M. hispida*, infects bacteria [14]. Further studies, with a greater number of coral colonies collected on different days and during different seasons, are needed to better understand the structure and specificity of the viral communities of these coral species. However, several sequences of the Gokushovirinae group were found in both libraries. Gokushoviruses belong to the Microviridae family and have been reported in several metagenomic studies, but they were never isolated and their hosts remain unknown [15]. Gokushovirus sequences comprised nearly 6% of the reads of the Sargasso Sea Virome (SARVir) [16], which can explain the similarities found between the *M. hispida*, *M. decactis*, and SARVir datasets. Labonte & Suttle [15] suggested that these viruses are highly virulent and can become dominant during a lytic event, because they have small genomes (around 4–5 kb) that permit rapid replication.

The way in which coral-bacteria associations are established is one of the main concerns of coral microbiology studies. Marine bacteria colonize the coral after larval settlement [17]. The assembly of microbial communities associated with marine organisms seems to be a process that is partly selective and partly random [18]. It is already well known that the microbial communities of corals are different from those of the surrounding environment [19], [20], [21], but there is little evidence concerning the functions required for the establishment and maintenance of a bacteria-coral association [22], [23], [24]. Comparative studies between coral microbial communities and other environments brings some insights into genes that could be advantageous for the coral niche. Comparison of the functional profiles of the present metagenomes with other metagenomes enabled the observation of some interesting features of the metagenomes of Brazilian corals, such as the relative enrichment of genes related to a colicin V production protein (CvpA), PF02674. The plasmid-borne cvpA gene encodes an inner membrane channel required for the production/secretion of the colicin V toxin in *E. coli* [25]. It has also been found in an aphid symbiont [26] and endosymbionts of deep-sea tubeworms [27]. *E. coli* cvpA mutants presented alterations in biofilm properties and curli fibers [28]. The glycosyltransferase-like family 2 (PF1011) includes putative proteins involved in capsule biosynthesis [29]. Glycosyltransferases are proteins responsible for the synthesis of polysaccharides or glycoproteins [30], and are related to the symbiotic colonization of *V. fischeri* [31], which has been suggested to be laterally transferred and advantageous in host gut [32]. Thus, the CvpA protein and glycosyltransferases are apparently involved in symbiotic relationships, and might act to enhance bacterial colonization of corals.

Comparisons of SEED functional profiles also revealed important functions in the metagenomes of the Brazilian corals. Genes that encode proteins involved in protein secretion systems type III (T3SS) and type IV (T4SS) were found to be enriched in our metagenomes. Protein secretion systems play a central role in modulating bacteria-host interactions. T3SS are found in pathogens and commensal bacteria that interact with both plant and animal hosts [33]. Several T3SS genes are encoded in pathogenicity islands or are located on plasmids, and are commonly subject to horizontal gene transfer [34]. T3SS has been frequently found in arthropoda-associated microbial communities [35]. Bondarev et al. [36] suggested that T3SS is involved in *Pseudovibrio* interactions with marine invertebrates. T4SS is able to transport not only protein but also DNA [33]. This system has been found enriched in microbial communities where HGT events are advantageous, because of intense selective pressure, and is often encoded on self-transmissible plasmids and integrative conjugative elements [35]. Based on this information, we suggest that these protein secretion systems can play a role in the establishment and tightening of bacterial interaction with the coral host, and for this reason they are subject to HGT and were found to be enhanced in the Brazilian coral metagenome.

In this study, most of the metagenome pairs shared relatively few identical sequences (254 of 465 comparisons resulted in J<1.0×10^−6^). The dataset pairs with highest J values included some metagenomes belonging to the same project and prepared by the same group, such as ArcticVir and GOMVir [16], SpongeAb1 and SpongeAb2 [37], and the two metagenomes sequenced in this work. In contrast, the metagenomes Gut_TS5 [38] and ChickenCecum [39] were sequenced by different groups. Given its nature, the analysis presented here may be influenced by the methodologies employed in different studies. However, we have found that metagenomes with the same sequencing methodology (cloning) or amplification methodology (MDA) do not share more sequences. Despite the limitations of the present results, some conclusions can be drawn that might help to guide future studies of identical nucleotide sequences that are shared between metagenomes. Network and node properties obtained from bacterial metagenome gene-sharing data can help to identify critical environments and factors for the swapping of genes among microbial communities. The betweenness centrality measure is defined as the frequency with which a node lies on the shortest path between two other network nodes, and a metagenome with a high betweenness value can transfer sequences to many other metagenomes in the network, with a low number of gene transfer events. In other words, it can function as a bridge between disconnected regions of the network [40]. Our results indicated that marine microbial communities may play this role. In epidemiologic terms, the marine environment presents higher degree of connectivity than the terrestrial one, and one of the reasons is the lack of dispersal barriers [41]. Therefore, micro-organisms such as bacteria and viruses can spread easily and rapid through oceans, which may explain the high betweenness values found in the Polynesia and WaterJF1 metagenomes. Viruses may be one of the most important carriers of genes through biomes, due to their ubiquity and abundance [42], [43]. Corroborating this notion, two studies have found a worldwide distribution of identical viral sequences [44], [45], showing that viruses can move between different environments.

Cluster or modularity analysis allows the identification of groups of nodes that share more sequences between them than with nodes outside the group. These groups can represent microbial communities with similar taxonomic or functional composition, or groups of communities where gene swapping is more likely to happen. In both cases, despite the impossibility of functional classification of the shared sequences, the metagenome clusters can be interpreted as groups of microbial communities that are under similar selective pressures. Examples are the cluster composed of microbial communities of the vertebrate digestive system (Gut_TS1, Gut_TS5, CowRumen, and ChickenCecum) and the one composed of marine viromes (GOMVir, ArcticVir, BBCVir, and TampaBay). The metagenomes primarily consisting of Atlantic Ocean phage sequences compose another cluster (*Mussismilia*, *Madracis*, and SARVir). It is interesting to note that the four coral metagenomes used in this study were not clustered together. One possible explanation for this is the fact that most of coral-derived microbial metagenomes also present host, algae, chloroplast and mitochondrial DNA, which make comparative analyses difficult [24].

Regarding the factors that influence the observed profile of shared sequences among datatsets, we have found that climate and habitat can influence the determination of metagenome groups; however, these influences seem to be slight, and other factors not considered in our analysis may be more important, such as physical-chemical and nutritional parameters, ocean and wind currents, and host biogeography, amongst others.

Finally, we consider that comparative metagenomics using a reference-independent approach is even better than using functional profiles (SEED and Pfam), because it led to a more clear discrimination of the microbial communities used in this study (Figures S1, S2 and S3). One explanation for the high overlapping among the datasets observed with SEED and Pfam profiles is that using reference-dependent approaches to compare metagenomes only takes in consideration the small amount of sequences that are similar to known sequences, and ignores the high amount of sequences from unknown organisms and with unknown function, which may be holding the differences among microbial communities.

## Conclusions

Comparative metagenomics allows the identification of microbial communities that share similar functional and taxonomic composition, and the observation of patterns controlling ecological niche partitioning. By comparing the metagenomes associated with two coral species with other metagenomes, we were able to identify functions that are probably either required or advantageous for coral colonization, such as transport proteins.

Besides of the simplicity of the reference-independent approach suggested in this work, the analysis of identical sequences shared among the microbial communities was found to be even better for the identification of groups of communities with similar niche requirements than the traditional analysis of functional profiles. This approach is not only helpful for the investigation of similarities between microbial communities, but also enables an indirect overview of gene exchange between communities. We consider this to be a successful procedure for use in comparative metagenomics, and we also suggest the use of tblastx comparisons in future studies.

## Supporting Information

Figure S1 **Visualization of Bray-Curtis similarities of Pfam profiles between metagenomes using non-metric multidimensional scaling.** Stress value  = 0.1048.
(TIF)

Figure S2 **Visualization of Bray-Curtis similarities of Level 3 (SEED subsystem) profiles between metagenomes using non-metric multidimensional scaling.** Stress value  = 0.1868.
(TIF)

Figure S3 **Visualization of Bray-Curtis similarities of gene-shared hits between metagenomes using non-metric multidimensional scaling.** Stress value  = 0.2484.
(TIF)

Table S1 **Information about the metagenomes utilized in this work.**
(DOCX)

Table S2 **Jaccard's similarities among the metagenomes obtained from gene-sharing data.**
(DOCX)

Table S3 **Prevalent Pfam domains in the shared sequences of four metagenome pairs.**
(DOCX)

Table S4 **Node centrality measures.**
(DOCX)

## References

[1] Dutilh BE, Schmieder R, Nulton J, Felts B, Salamon P, et al (2012) Reference-independent comparative metagenomics using cross-assembly: crAss. Bioinformatics 28: 3225–3231. 23074261 10.1093/bioinformatics/bts613 PMC3519457

[2] Teeling H, Glöckner FO (2012) Current opportunities and challenges in microbial metagenome analysis - a bioinformatic perspective. Brief Bioinform 13: 728–742. 22966151 10.1093/bib/bbs039 PMC3504927

[3] Kloesges T, Popa O, Martin W, Dagan T (2010) Networks of gene sharing among 329 proteobacterial genomes reveal differences in lateral gene transfer frequency at different phylogenetic depths. Mol Biol Evol 28: 1057–1074. 21059789 10.1093/molbev/msq297 PMC3021791

[4] Smillie CS, Smith MB, Friedman J, Cordero OX, David LA, et al (2011) Ecology drives a global network of gene exchange connecting the human microbiome. Nature 480: 241–244. 22037308 10.1038/nature10571

[5] Forsberg KJ, Reyes A, Wang B, Selleck EM, Sommer MO, et al (2012) The shared antibiotic resistome of soil bacteria and human pathogens. Science 337: 1107–1111. 22936781 10.1126/science.1220761 PMC4070369

[6] Schmieder R, Edwards R (2011) Quality control and preprocessing of metagenomic datasets. Bioinformatics 27: 863–86.. 21278185 10.1093/bioinformatics/btr026 PMC3051327

[7] Schmieder R, Lim YW, Rohwer F, Edwards R (2010) TagCleaner: Identification and removal of tag sequences from genomic and metagenomic datasets. *BMC* Bioinformatics 11: 341. 20573248 10.1186/1471-2105-11-341 PMC2910026

[8] Meyer F, Paarmann D, D'Souza M, Olson R, Glass EM, et al (2008) The metagenomics RAST server - a public resource for the automatic phylogenetic and functional analysis of metagenomes. BMC Bioinformatics 9: 386. 18803844 10.1186/1471-2105-9-386 PMC2563014

[9] Lingner T, Asshauer KP, Schreiber F, Meinicke P (2011) CoMet - a web server for comparative functional profiling of metagenomes. Nucleic Acids Res 39: W518–523. 21622656 10.1093/nar/gkr388 PMC3125781

[10] Roux S, Faubladier M, Mahul A, Paulhe N, Bernard A, et al (2011) Metavir: a web server dedicated to virome analysis. Bioinformatics 27: 3074–3075. 21911332 10.1093/bioinformatics/btr519

[11] White JR, Nagarajan N, Pop M (2009) Statistical methods for detecting differentially abundant features in clinical metagenomic samples. PLOS Comput Biol 5: e1000352. 19360128 10.1371/journal.pcbi.1000352 PMC2661018

[12] Szalay-Beko M, Palotai R, Szappanos B, Kovács IA, Papp B, et al (2012) ModuLand plug-in for Cytoscape: determination of hierarchical layers of overlapping network modules and community centrality. Bioinformatics 28: 2202–2204. 22718784 10.1093/bioinformatics/bts352

[13] Delwart E, Li L (2012) Rapidly expanding genetic diversity and host range of the Circoviridae viral family and other Rep encoding small circular ssDNA genomes. Virus Res 164: 114–121. 22155583 10.1016/j.virusres.2011.11.021 PMC3289258

[14] Krupovic M, Forterre P (2011) Microviridae goes temperate: microvirus-related proviruses reside in the genomes of Bacteroidetes. PLOS One 10: e19893. 10.1371/journal.pone.0019893 PMC3091885 21572966

[15] Labonté JM, Suttle CA (2013) Metagenomic and whole-genome analysis reveals new lineages of gokushoviruses and biogeographic separation in the sea. Front Microbiol 4: 404. 24399999 10.3389/fmicb.2013.00404 PMC3871881

[16] Angly FE, Felts B, Breitbart M, Salamon P, Edwards RA, et al (2006) The marine viromes of four oceanic regions. PLOS Biol 4: e368. 17090214 10.1371/journal.pbio.0040368 PMC1634881

[17] Sharp KH, Ritchie KB, Schupp PJ, Ritson-Williams R, Paul VJ (2010) Bacterial acquisition in juveniles of several broadcast spawning coral species. PLOS One 5: e10898. 20526374 10.1371/journal.pone.0010898 PMC2878338

[18] Burke C, Steinberg P, Rusch D, Kjelleberg S, Thomas T (2011) Bacterial community assembly based on functional genes rather than species. Proc Natl Acad Sci USA 108: 14288–93. 21825123 10.1073/pnas.1101591108 PMC3161577

[19] Carlos C, Torres TT, Ottoboni LM (2013) Bacterial communities and species-specific associations with the mucus of Brazilian coral species. Sci Rep 2013: 31624. 10.1038/srep01624 PMC3620669 23567936

[20] Schöttner S, Wild C, Hoffmann F, Boetius A, Ramette A (2012) Spatial scales of bacterial diversity in cold-water coral reef ecosystems. PLOS One 7: e32093. 22403625 10.1371/journal.pone.0032093 PMC3293894

[21] Schöttner S, Hoffmann F, Wild C, Rapp HT, Boetius A, et al (2009) Inter- and intra-habitat bacterial diversity associated with cold-water corals. ISME J 3: 156–759. 10.1038/ismej.2009.15 19279671

[22] Garcia GD, Gregoracci GB, Santos Ede O, Meirelles PM, Silva GG, et al (2013) Metagenomic analysis of healthy and white plague-affected Mussismilia braziliensis corals. Microb Ecol 65: 1076–1086. 23314124 10.1007/s00248-012-0161-4

[23] Vega Thurber R, Willner-Hall D, Rodriguez-Mueller B, Desnues C, Edwards RA, et al (2009) Metagenomic analysis of stressed coral holobionts. Environ Microbiol 11: 2148–2163. 19397678 10.1111/j.1462-2920.2009.01935.x

[24] Wegley L, Edwards R, Rodriguez-Brito B, Liu H, Rohwer F (2007) Metagenomic analysis of the microbial community associated with the coral Porites astreoides. Environ Microbiol 9: 2707–2719. 17922755 10.1111/j.1462-2920.2007.01383.x

[25] Fath MJ, Mahanty HK, Kolter R (1989) Characterization of a purF operon mutation which affects colicin V production. J Bacteriol 171: 3158–3161.. 2542219 10.1128/jb.171.6.3158-3161.1989 PMC210030

[26] Charles H, Balmand S, Lamelas A, Cottret L, Pérez-Brocal V, et al (2011) A genomic reappraisal of symbiotic function in the aphid/*Buchnera* symbiosis: reduced transporter sets and variable membrane organisations. PLOS One 6: e29096. 22229056 10.1371/journal.pone.0029096 PMC3246468

[27] Gardebrecht A, Markert S, Sievert SM, Felbeck H, Thürmer A, et al (2012) Physiological homogeneity among the endosymbionts of *Riftia pachyptila* and *Tevnia jerichonana* revealed by proteogenomics. ISME J 6: 766–776. 22011719 10.1038/ismej.2011.137 PMC3309349

[28] Hadjifrangiskou M, Gu AP, Pinkner JS, Kostakioti M, Zhang EW, et al (2012) Transposon mutagenesis identifies uropathogenic *Escherichia coli* biofilm factors. J Bacteriol 2012 194: 6195–6205. 10.1128/JB.01012-12 PMC3486386 22984258

[29] Breton C (2006) Structures and mechanisms of glycosyltransferases. Glycobiology 16: 29–37. 16037492 10.1093/glycob/cwj016

[30] Upreti RK, Kumar M, Shankar V (2003) Bacterial glycoproteins: functions, biosynthesis and applications. Proteomics 3: 363–379. 12687605 10.1002/pmic.200390052

[31] Yip ES, Grublesky BT, Hussa EA, Visick KL (2005) A novel conserved cluster of genes promotes symbiotic colonization and sigma-dependent biofilm formation by *Vibrio fischeri* . Mol Microbiol 57: 1485–1498. 16102015 10.1111/j.1365-2958.2005.04784.x

[32] Brown Kav A, Sasson G, Jami E, Doron-Faigenboim A, Benhar I, et al (2012) Insights into the bovine rumen plasmidome. Proc Natl Acad Aci USA 109: 5452–5457. 10.1073/pnas.1116410109 PMC3325734 22431592

[33] Tseng TT, Tyler BM, Setubal JC (2009) Protein secretion systems in bacterial-host associations, and their description in the Gene Ontology. BMC Microbiol 9: S2. 19278550 10.1186/1471-2180-9-S1-S2 PMC2654662

[34] Gophna U, Ron EZ, Graur D (2003) Bacterial type III secretion systems are ancient and evolved by multiple horizontal-transfer events. Gene 312: 151–63. 12909351 10.1016/s0378-1119(03)00612-7

[35] Barret M, Egan F, O'Gara F (2013) Distribution and diversity of bacterial secretion systems across metagenomic datasets. Environ Microbiol 5: 117–126. 10.1111/j.1758-2229.2012.00394.x 23757140

[36] Bondarev V, Richter M, Romano S, Piel J, Schwedt A, et al (2013) The genus *Pseudovibrio* contains metabolically versatile bacteria adapted for symbiosis. Environ Microbiol 15: 2095–2113. 23601235 10.1111/1462-2920.12123 PMC3806328

[37] Trindade-Silva AE, Rua C, Silva GG, Dutilh BE, Moreira AP, et al (2012) Taxonomic and functional microbial signatures of the endemic marine sponge *Arenosclera brasiliensis* . PLOS One 7: e39905. 22768320 10.1371/journal.pone.0039905 PMC3388064

[38] Turnbaugh PJ, Hamady M, Yatsunenko T, Cantarel BL, Duncan A, et al (2009) A core gut microbiome in obese and lean twins. Nature 457: 480–484. 19043404 10.1038/nature07540 PMC2677729

[39] Qu A, Brulc JM, Wilson MK, Law BF, Theoret JR, et al (2008) Comparative metagenomics reveals host specific metavirulomes and horizontal gene transfer elements in the chicken cecum microbiome. PLoS One 3: e2945. 18698407 10.1371/journal.pone.0002945 PMC2492807

[40] Tamminen M, Virta M, Fani R, Fondi M (2011) Large-scale analysis of plasmid relationships through gene-sharing networks. Mol Biol Evol 29: 1225–1240. 22130968 10.1093/molbev/msr292

[41] McCallum H, Harvell D, Dobson A (2003) Rates of spread of marine pathogens. Ecol Lett 6: 1062–1067.

[42] Rohwer F, Thurber RV (2009) Viruses manipulate the marine environment. Nature 459: 207–212. 19444207 10.1038/nature08060

[43] Muniesa M, Colomer-Lluch M, Jofre J (2013) Potential impact of environmental bacteriophages in spreading antibiotic resistance genes. Future Microbiol 8: 739–751. 23701331 10.2217/fmb.13.32

[44] Breitbart M, Miyake JH, Rohwer F (2004) Global distribution of nearly identical phage-encoded DNA sequences. FEMS Microbiol Lett 236: 249–256. 15251204 10.1016/j.femsle.2004.05.042

[45] Short CM, Suttle CA (2005) Nearly identical bacteriophage structural gene sequences are widely distributed in both marine and freshwater environments. Appl Environ Microbiol 71: 480–486. 15640224 10.1128/AEM.71.1.480-486.2005 PMC544240

