# Supplementary figures and images for: Comparative Metagenomic Analysis of Coral Microbial Communities Using a Reference-Independent Approach
Source: PLoS One. 2014 Nov 7;9(11):e111626. doi: 10.1371/journal.pone.0111626 (PMC4224422; doi:10.1371/journal.pone.0111626)

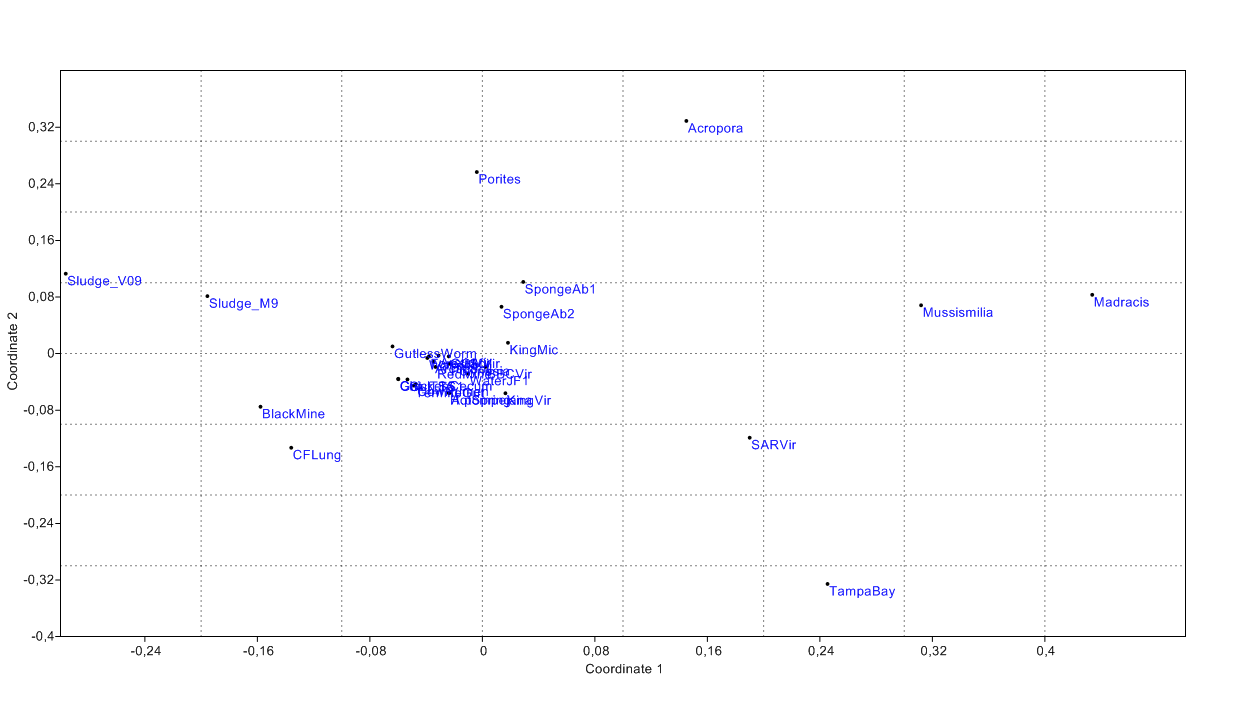

Supplement: Figure S1 — Visualization of Bray-Curtis similarities of Pfam profiles between metagenomes using non-metric multidimensional scaling. Stress value = 0.1048. (TIF) [file pone.0111626.s001.tif]

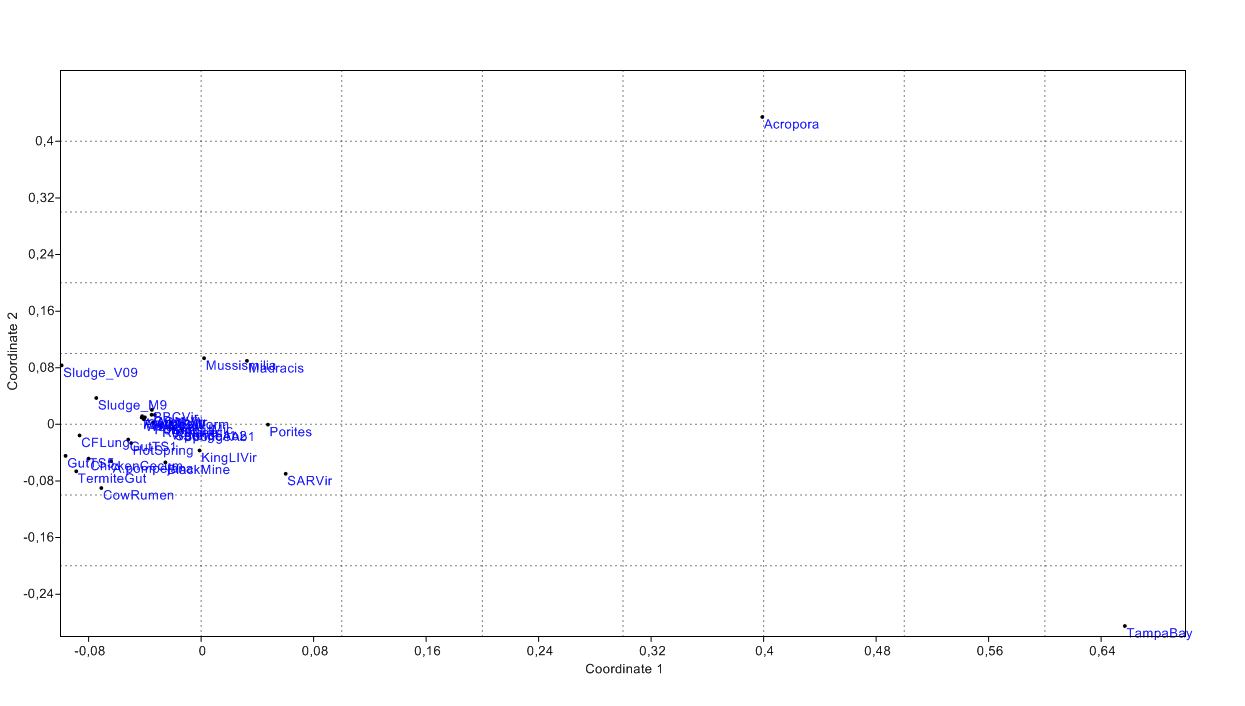

Supplement: Figure S2 — Visualization of Bray-Curtis similarities of Level 3 (SEED subsystem) profiles between metagenomes using non-metric multidimensional scaling. Stress value = 0.1868. (TIF) [file pone.0111626.s002.tif]

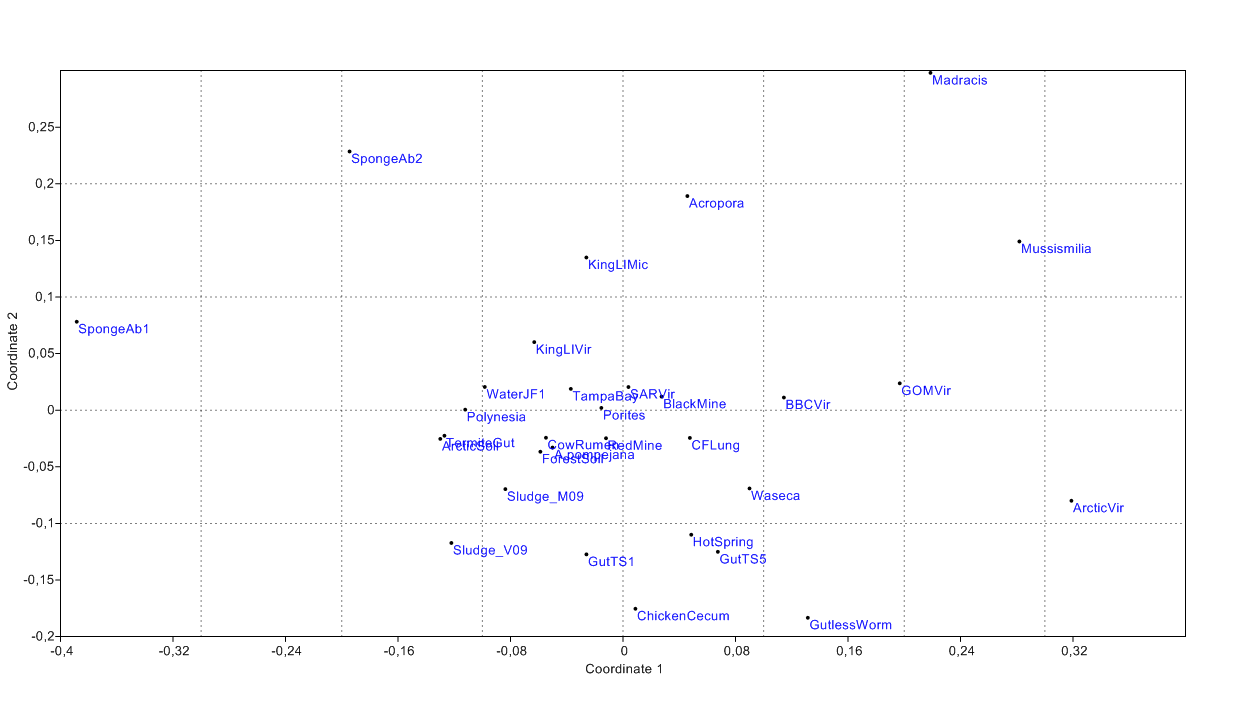

Supplement: Figure S3 — Visualization of Bray-Curtis similarities of gene-shared hits between metagenomes using non-metric multidimensional scaling. Stress value = 0.2484. (TIF) [file pone.0111626.s003.tif]
