# Supplementary material for: Comparative Metagenomic Analysis of Coral Microbial Communities Using a Reference-Independent Approach
Source: PLoS One. 2014 Nov 7;9(11):e111626. doi: 10.1371/journal.pone.0111626 (PMC4224422; doi:10.1371/journal.pone.0111626)
Supplement: Table S1 — Information about the metagenomes utilized in this work. (DOCX) [file pone.0111626.s004.docx]

| Metagenome | MG-RAST ID | Number of sequences | Average read length | % of annotated reads with SEED subsystems | tRNA (%) | Average GC (%) | TaxyPro classification | | | | Pfam hits | Latitude | Longitude | Environment | Lifestyle | Sample Origin | Sample Type | Climate | MDA | Cloning | Reference |
| --- | --- | --- | --- | --- | --- | --- | --- | --- | --- | --- | --- | --- | --- | --- | --- | --- | --- | --- | --- | --- | --- |
|  |  |  |  |  |  |  | Bacteria | Eukaryota | VMG | Viruses |  |  |  |  |  |  |  |  |  |  |  |
| A.pompejana | 4441102.3 | 293065 | 990 | 45.5 | 0.065 | 39.62 | 90.5 | 3.4 | 4.8 | 0.09 | 7443 | 9.84 | -104.28 | Marine | Host-associated | Thermotolerant polychaete | Microbial | Temperate | no | yes | [1] |
| Acropora | 4445755.3 | 403686 | 344 | 3.6 | 0.000 | 39.91 | 13.6 | 60.1 | 20.7 | 4.7 | 6279 | -19.1 | 146.5 | Marine | Host-associated | Coral | Microbial | Tropical | yes | no | [2] |
| ArcticVir | 4440306.3 | 611026 | 100 | 27.6 | 0.044 | 62.2 | 98.7 | 0.3 | 0.7 | 0.05 | 3719 | 71.34 | 128.19 | Marine | Free-living | Seawater | Viral | Polar | yes | no | [3] |
| ArcticSoil | 4450125.3 | 98848 | 413 | 54 | 0.058 | 62.00 | 97 | 1.4 | 0.8 | 0.08 | 2673 | 80.6 | 13.48 | Terrestrial | Free-living | Peat Soil | Microbial | Polar | no | no | [4] |
| BBCVir | 4440305.3 | 368025 | 103 | 4.1 | 0.01 | 47.04 | 68.9 | 0.9 | 28.2 | 1.1 | 2008 | 32.53 | 97.51 | Marine | Free-living | Seawater | Viral | Temperate | yes | no | [3] |
| BlackMine | 4440282.3 | 248038 | 101 | 0 | 0.029 | 44.72 | 35 | 14.5 | 47 | 0.5 | 1525 | 47.81 | -92.24 | Terrestrial | Free-living | Mine Drainage | Microbial | Temperate | yes | no | [5] |
| CFLung | 4440051.3 | 28171 | 83 | 0 | 0.127 | 39.78 | 34 | 4 | 61.3 | 0.1 | 2323 | 32.87 | -117.1 | Terrestrial | Host-associated | Sputum | Viral | Temperate | yes | no | [6] |
| ChickenCecum | 4440283.3 | 272236 | 105 | 41.3 | 0.078 | 46.71 | 98.7 | 0.6 | 0.4 | 0.1 | 2755 | 40.11 | -88.2 | Terrestrial | Host-associated | Ceca | Microbial | Temperate | no | no | [7] |
| CowRumen | 4441679.3 | 23805 | 102 | 20.8 | 0.285 | 51.47 | 97.2 | 0.6 | 1 | 0.02 | 2975 | 40.5 | -88.99 | Terrestrial | Host-associated | Whole rumen digesta | Plasmidial | Temperate | yes | no | [8] |
| ForestSoil | 4446153.3 | 642197 | 435 | 43.6 | 0.066 | 59.83 | 96.3 | 1.2 | 1.2 | 0.03 | 3957 | 18.3 | -65.83 | Terrestrial | Free-living | Bisley watershed | Microbial | Tropical | no | no | Not found |
| GOMVir | 4440304.3 | 222659 | 102 | 9.5 | 0.018 | 49.01 | 90.4 | 0.2 | 8.4 | 0.5 | 2843 | 26.09 | 88.73 | Marine | Free-living | Seawater | Viral | Temperate | yes | no | [3] |
| Gut_TS1 | 4440452.7 | 313773 | 1003 | 0 | 0.108 | 50.88 | 82.1 | 4.2 | 9.7 | 0.07 | 1496 | 42.8 | 10.14 | Terrestrial | Host-associated | Faecal | Microbial | Temperate | no | no | [9] |
| Gut_TS5 | 4440461.5 | 192843 | 249 | 53.8 | 0.022 | 54.3 | 98.6 | 0.3 | 0.5 | 0.06 | 5219 | 38.64 | -90.3 | Terrestrial | Host-associated | Faecal | Microbial | Temperate | no | no | [9] |
| GutlessWorm | 4441115.3 | 429846 | 212 | 56.6 | 0.104 | 42.88 | 99.3 | 0.1 | 0.3 | 0.04 | 3201 | 38.63 | -90.25 | Marine | Host-associated | Gutless worm | Microbial | Temperate | no | yes | [10] |
| HotSpring | 4460449.3 | 685935 | 581 | 61.3 | 0.097 | 36.5 | 95.9 | 0.8 | 2.7 | 0.1 | 4017 | 40.69 | -111.2 | Terrestrial | Free-living | Biofilm mat | Microbial | Temperate | no | no | Not found |
| KingLIMic | 4440037.3 | 143977 | 105 | 1.8 | 0.01 | 46.96 | 62.1 | 21.6 | 9.5 | 4.2 | 8031 | 6.38 | -162.33 | Marine | Free-living | Seawater | Microbial | Tropical | yes | no | [11] |
| KingLIVir | 4440036.3 | 79503 | 109 | 6.5 | 0.011 | 37.91 | 75.6 | 1.6 | 18 | 3.4 | 4240 | 6.38 | -162.33 | Marine | Free-living | Seawater | Viral | Tropical | yes | no | [11] |
| Madracis | 4516541.3 | 29358 | 513 | 3.3 | 0.064 | 45.18 | 11.5 | 7.1 | 72.4 | 8.3 | 3281 | -23.84 | -45.16 | Marine | Host-associated | Coral | Microbial | Tropical | yes | no | This work |
| Mussismilia | 4516694.3 | 368772 | 482 | 4.4 | 0.002 | 43.98 | 12 | 19.2 | 62.4 | 5.9 | 3104 | -23.84 | -45.16 | Marine | Host-associated | Coral | Microbial | Tropical | yes | no | This work |
| Polynesia | 4441167.3 | 46267 | 1075 | 68.3 | 0.469 | 51.3 | 84.2 | 1.9 | 3.2 | 0.8 | 4031 | -17.47 | -149.812 | Marine | Free-living | Coral | Microbial | Tropical | no | no | [12] |
| Porites | 4440319.3 | 266015 | 103 | 5.5 | 0.001 | 48.05 | 6.8 | 82.5 | 8.2 | 0.7 | 3334 | 9.33 | -82.84 | Marine | Host-associated | Coral | Microbial | Tropical | yes | no | [13] |
| RedMine | 4440281.3 | 27582 | 107 | 14.7 | 0.066 | 49.6 | 96.7 | 0.5 | 1.8 | 0.1 | 4664 | 47.81 | -92.24 | Terrestrial | Free-living | Mine Drainage | Microbial | Temperate | yes | no | [5] |
| SARVir | 4440322.3 | 357238 | 105 | 5.4 | 0.006 | 38.76 | 19.3 | 1.1 | 73.5 | 5 | 3624 | 32.16 | -64.5 | Marine | Free-living | Seawater | Viral | Temperate | yes | no | [3] |
| Sludge_M9 | 4464073.3 | 166658 | 270 | 28.3 | 0.025 | 52.67 | 49.8 | 0.5 | 45.7 | 3.2 | 4062 | 46.51 | 6.51 | Terrestrial | Free-living | Activated Sludge | Plasmidial | Temperate | no | no | [14] |
| Sludge_V09 | 4464109.3 | 325908 | 335 | 7.1 | 0.009 | 62.14 | 56.9 | 0.1 | 30.5 | 11.2 | 2289 | 46.3 | 7.85 | Terrestrial | Free-living | Activated Sludge | Plasmidial | Temperate | no | no | [14] |
| SpongeAb1 | 4461456.3 | 56401 | 259 | 7.4 | 0.005 | 43.51 | 22 | 62.2 | 12.8 | 0.2 | 3061 | -22.73 | -41.87 | Marine | Host-associated | Sponge | Microbial | Tropical | no | no | [15] |
| SpongeAb2 | 4461455.3 | 142408 | 279 | 9.1 | 0.018 | 44.09 | 30.8 | 50.9 | 14.9 | 0.4 | 3256 | -22.73 | -41.87 | Marine | Host-associated | Sponge | Microbial | Tropical | no | no | [15] |
| TampaBay | 4440102.3 | 257075 | 105 | 5 | 0.006 | 39.25 | 0.2 | 0.3 | 79 | 20.2 | 2169 | 37.62 | -122.07 | Marine | Free-living | Concentrated marine microbial community | Viral | Temperate | yes | no | [16] |
| TermiteGut | 4442701.3 | 57641 | 1067 | 69.9 | 0.053 | 48.26 | 94.3 | 2.3 | 2.3 | 0.1 | 3742 | 10.11 | -83.51 | Terrestrial | Host-associated | Termite Gut | Microbial | Tropical | no | yes | [17] |
| Waseca | 4441091.3 | 138347 | 1116 | 0 | 0.118 | 57.34 | 87 | 2.6 | 4.6 | 2.3 | 6467 | 43.96 | 93.66 | Terrestrial | Free-living | Agricultural Soil | Microbial | Temperate | no | yes | [18] |
| WaterJF1 | 4453374.3 | 85742 | 435 | 34.7 | 0.141 | 41.67 | 74.9 | 4.8 | 9.6 | 7.4 | 6778 | -22.73 | -41.87 | Marine | Free-living | Seawater | Microbial | Tropical | no | no | [15] |

**References: 1.** Grzymski JJ, Murray AE, Campbell BJ, Kaplarevic M, Gao GR, et al (2008) Metagenome analysis of an extreme microbial symbiosis reveals eurythermal adaptation and metabolic flexibility. Proc Natl Acad Sci USA 105: 17516-21. **2** Littman R, Willis BL, Bourne DG (2011) Metagenomic analysis of the coral holobiont during a natural bleaching event on the Great Barrier Reef. Environ Microbiol Rep 3: 651-60. **3.** Angly FE, Felts B, Breitbart M, Salamon P, Edwards RA, et al. (2006) The marine viromes of four oceanic regions. PLOS Biol 4: e368. **4.** Tveit A, Schwacke R, Svenning MM, Urich T (2013) Organic carbon transformations in high-Arctic peat soils: key functions and microorganisms. ISME J 7: 299-311; **5.** Edwards RA, Rodriguez-Brito B, Wegley L, Haynes M, Breitbart M, et al. (2006) Using pyrosequencing to shed light on deep mine microbial ecology. BMC Genomics 20: 57. **6.** Willner D, Furlan M, Haynes M, Schmieder R, Angly FE, et al (2009) Metagenomic analysis of respiratory tract DNA viral communities in cystic fibrosis and non-cystic fibrosis individuals. PLoS One 4: e7370. **7.** Qu A, Brulc JM, Wilson MK, Law BF, Theoret JR, et al. (2008) Comparative metagenomics reveals host specific metavirulomes and horizontal gene transfer elements in the chicken cecum microbiome. PLoS One 3: e2945. **8.** Brown Kav A, Sasson G, Jami E, Doron-Faigenboim A, Benhar I, et al. (2012) Insights into the bovine rumen plasmidome. Proc Natl Acad Aci USA 109: 5452-5457. **9.** Turnbaugh PJ, Hamady M, Yatsunenko T, Cantarel BL, Duncan A, et al. (2009) A core gut microbiome in obese and lean twins. Nature 457: 480-484. **10.** Woyke T, Teeling H, Inanova NN, Huntemann M, Richter M, et al (2006) Symbiosis insights through metagenomic analysis of a microbial consortium. Nature 443: 950-955. **11.** Dinsdale EA, Pantos O, Smriga S, Edwards RA, Angly F, et al. (2008) Microbial ecology of four coral atolls in the Northern Line Islands. PLoS One 3: e1584. **12.** Rusch DB, Halpern AL, Sutton G, Heidelberg KB, Williamson S, et al. (2007) The Sorcerer II Global Ocean Sampling expedition: northwest Atlantic through eastern tropical Pacific. PLoS Biol 5: e77. **13.** Wegley L, Edwards R, Rodriguez-Brito B, Liu H, Rohwer F (2007) Metagenomic analysis of the microbial community associated with the coral Porites astreoides. Environ Microbiol 9: 2707-2719. **14.** Sentchilo V, Mayer AP, Guy L, Miyazaki R, Green Tringe S, et al. (2013) Community-wide plasmid gene mobilization and selection. ISME J 7: 1173-86. **15.** Trindade-Silva AE, Rua C, Silva GG, Dutilh BE, Moreira AP, et al. (2012) Taxonomic and functional microbial signatures of the endemic marine sponge Arenosclera brasiliensis. PLOS One 7: e39905. **16.** McDaniel L, Breitbart M, Mobberley J, Long A, Haynes M, et al. (2008) Metagenomic analysis of lysogeny in Tampa Bay: implications for prophage gene expression. PLoS One 3: e3263. **17.** Warnecke F, Luginbühl P, Ivanova N, Ghassemian M, Richardson TH, et al. (2007) Metagenomic and functional analysis of hindgut microbiota of a wood-feeding higher termite. Nature 450: 560-565. **18.** Tringe SG, von Mering C, Kobayashi A, Salamov AA, Chen K, et al (2005) Comparative metagenomics of microbial communities. Science 308: 554-557.
