# Supplementary material for: Comparative Metagenomic Analysis of Coral Microbial Communities Using a Reference-Independent Approach
Source: PLoS One. 2014 Nov 7;9(11):e111626. doi: 10.1371/journal.pone.0111626 (PMC4224422; doi:10.1371/journal.pone.0111626)
Supplement: Table S2 — Jaccard's similarities among the metagenomes obtained from gene-sharing data. (DOCX) [file pone.0111626.s005.docx]

|  | A.pompejana | Acropora | ArcticSoil | ArcticVir | BBCVir | BlackMine | CFLung | ChickenCecum | CowRumen | ForestSoil | GOMVir | Gut_TS1 | Gut_TS5 | GutlessWorm | HotSpring | KingLIMic | KingLIVir | Madracis | Mussismilia | Polynesia | Porites | RedMine | SARVir | Sludge_M09 | Sludge_V09 | SpongeAb1 | SpongeAb2 | TampaBay | TermiteGut | Waseca | WaterJF1 |
| --- | --- | --- | --- | --- | --- | --- | --- | --- | --- | --- | --- | --- | --- | --- | --- | --- | --- | --- | --- | --- | --- | --- | --- | --- | --- | --- | --- | --- | --- | --- | --- |
| A.pompejana | 1 | 0.000000035116752 | 0.000000012076812 | 0.000000000000000 | 0.000000000000000 | 0.000000042597155 | 0.000000000000000 | 0.000000019269460 | 0.000000000000000 | 0.000000000000000 | 0.000000022943883 | 0.000000000000000 | 0.000000087096512 | 0.000023003912541 | 0.000000059468549 | 0.000000000000000 | 0.000000000000000 | 0.000000031737689 | 0.000000042713457 | 0.000000196981166 | 0.000000120329046 | 0.000000450755807 | 0.000000000000000 | 0.000000021051320 | 0.000000191567901 | 0.000000160817089 | 0.000000512481168 | 0.000000000000000 | 0.000000039786245 | 0.000008767052831 | 0.000000024412964 |
| Acropora | 0.000000035116752 | 1 | 0.000000000000000 | 0.000000000000000 | 0.000000000000000 | 0.000000547537611 | 0.000000088208335 | 0.000000000000000 | 0.000000000000000 | 0.000000000000000 | 0.000000079210394 | 0.000000000000000 | 0.000000000000000 | 0.000000000000000 | 0.000000001866218 | 0.000021102384509 | 0.000000292066347 | 0.000000006956063 | 0.000001455633836 | 0.000000016082592 | 0.000000207936366 | 0.000000000000000 | 0.000000000000000 | 0.000000000000000 | 0.000000000000000 | 0.000000000000000 | 0.000000000000000 | 0.000000000000000 | 0.000000000000000 | 0.000000000000000 | 0.000000005743898 |
| ArcticSoil | 0.000000012076812 | 0.000000000000000 | 1 | 0.000000026629341 | 0.000000000000000 | 0.000000000000000 | 0.000000000000000 | 0.000000000000000 | 0.000000000000000 | 0.000000009368517 | 0.000000000000000 | 0.000000000000000 | 0.000000000000000 | 0.000000016873641 | 0.000000038644286 | 0.000000000000000 | 0.000000083560297 | 0.000000010439195 | 0.000000027434058 | 0.000000000000000 | 0.000000034506606 | 0.000000047410076 | 0.000000000000000 | 0.000000024099157 | 0.000000000000000 | 0.000000181278552 | 0.000000299110323 | 0.000000000000000 | 0.000000000000000 | 0.000000010239761 | 0.000000012793536 |
| ArcticVir | 0.000000000000000 | 0.000000000000000 | 0.000000026629341 | 1 | 0.000136143662879 | 0.000000101881590 | 0.000000053377003 | 0.000000000000000 | 0.000000000000000 | 0.000000006376665 | 0.000312836269899 | 0.000000000000000 | 0.000000000000000 | 0.000000000000000 | 0.000000000000000 | 0.000000000000000 | 0.000000000000000 | 0.000000000000000 | 0.000000000000000 | 0.000000166615260 | 0.000000000000000 | 0.000000105820431 | 0.000000000000000 | 0.000000000000000 | 0.000000241789733 | 0.000000000000000 | 0.000000000000000 | 0.000000000000000 | 0.000000000000000 | 0.000000058281480 | 0.000000000000000 |
| BBCVir | 0.000000000000000 | 0.000000000000000 | 0.000000000000000 | 0.000136143662879 | 1 | 0.000000049906834 | 0.000000000000000 | 0.000000000000000 | 0.000000000000000 | 0.000000000000000 | 0.000041768605825 | 0.000000000000000 | 0.000000000000000 | 0.000000000000000 | 0.000000000000000 | 0.000000000000000 | 0.000000000000000 | 0.000001522676306 | 0.000001550757939 | 0.000000053316266 | 0.000000000000000 | 0.000000041930551 | 0.000000037706302 | 0.000000029668888 | 0.000000182348458 | 0.000000000000000 | 0.000000000000000 | 0.000000471965009 | 0.000000000000000 | 0.000000000000000 | 0.000000000000000 |
| BlackMine | 0.000000042597155 | 0.000000547537611 | 0.000000000000000 | 0.000000101881590 | 0.000000049906834 | 1 | 0.000018828243323 | 0.000000000000000 | 0.000000000000000 | 0.000000000000000 | 0.000000169299809 | 0.000000000000000 | 0.000000000000000 | 0.000016692173386 | 0.000000524304972 | 0.000000000000000 | 0.000000000000000 | 0.000000000000000 | 0.000000000000000 | 0.000000108400693 | 0.000000000000000 | 0.000035812905436 | 0.000000000000000 | 0.000001369336154 | 0.000000288856662 | 0.000000000000000 | 0.000000000000000 | 0.000000000000000 | 0.000000052409803 | 0.000001370441446 | 0.000000038361261 |
| CFLung | 0.000000000000000 | 0.000000088208335 | 0.000000000000000 | 0.000000053377003 | 0.000000000000000 | 0.000018828243323 | 1 | 0.000000232871530 | 0.000000133287632 | 0.000000000000000 | 0.000000000000000 | 0.000000457338663 | 0.000000461711355 | 0.000000041582135 | 0.000000052255458 | 0.000000000000000 | 0.000000000000000 | 0.000000000000000 | 0.000000000000000 | 0.000000027892978 | 0.000000102409776 | 0.000000045068856 | 0.000000000000000 | 0.000000109222587 | 0.000000000000000 | 0.000000000000000 | 0.000000000000000 | 0.000000000000000 | 0.000000000000000 | 0.000000011336846 | 0.000000033747682 |
| ChickenCecum | 0.000000019269460 | 0.000000000000000 | 0.000000000000000 | 0.000000000000000 | 0.000000000000000 | 0.000000000000000 | 0.000000232871530 | 1 | 0.000002952893636 | 0.000000000000000 | 0.000000000000000 | 0.000141196479878 | 0.000346694484037 | 0.000000011913822 | 0.000000040469437 | 0.000000000000000 | 0.000000000000000 | 0.000000000000000 | 0.000000000000000 | 0.000000098920259 | 0.000000000000000 | 0.000000000000000 | 0.000000000000000 | 0.000000902602193 | 0.000000000000000 | 0.000000000000000 | 0.000000000000000 | 0.000000000000000 | 0.000000000000000 | 0.000000034192446 | 0.000000102872252 |
| CowRumen | 0.000000000000000 | 0.000000000000000 | 0.000000000000000 | 0.000000000000000 | 0.000000000000000 | 0.000000000000000 | 0.000000133287632 | 0.000002952893636 | 1 | 0.000000000000000 | 0.000000000000000 | 0.000002688586582 | 0.000005245421151 | 0.000000000000000 | 0.000000012058630 | 0.000000000000000 | 0.000000000000000 | 0.000000000000000 | 0.000000000000000 | 0.000000030611560 | 0.000000000000000 | 0.000000052619526 | 0.000000000000000 | 0.000000051973728 | 0.000000000000000 | 0.000000000000000 | 0.000000000000000 | 0.000000000000000 | 0.000000000000000 | 0.000000023522759 | 0.000000000000000 |
| ForestSoil | 0.000000000000000 | 0.000000000000000 | 0.000000009368517 | 0.000000006376665 | 0.000000000000000 | 0.000000000000000 | 0.000000000000000 | 0.000000000000000 | 0.000000000000000 | 1 | 0.000000000000000 | 0.000000000000000 | 0.000000000000000 | 0.000000005049462 | 0.000000005895807 | 0.000000000000000 | 0.000000000000000 | 0.000000000000000 | 0.000000000000000 | 0.000000009114601 | 0.000000000000000 | 0.000000000000000 | 0.000000000000000 | 0.000000012440561 | 0.000000015554746 | 0.000000000000000 | 0.000000009410985 | 0.000000000000000 | 0.000000000000000 | 0.000000004609830 | 0.000000000000000 |
| GOMVir | 0.000000022943883 | 0.000000079210394 | 0.000000000000000 | 0.000312836269899 | 0.000041768605825 | 0.000000169299809 | 0.000000000000000 | 0.000000000000000 | 0.000000000000000 | 0.000000000000000 | 1 | 0.000000000000000 | 0.000000000000000 | 0.000000006070482 | 0.000000343170265 | 0.000000000000000 | 0.000000183919986 | 0.000000229656339 | 0.000000000000000 | 0.000000046524075 | 0.000000000000000 | 0.000000000000000 | 0.000000494114566 | 0.000000000000000 | 0.000000074334168 | 0.000000000000000 | 0.000000000000000 | 0.000007039026661 | 0.000000026236696 | 0.000010378494289 | 0.000001095238900 |
| Gut_TS1 | 0.000000000000000 | 0.000000000000000 | 0.000000000000000 | 0.000000000000000 | 0.000000000000000 | 0.000000000000000 | 0.000000457338663 | 0.000141196479878 | 0.000002688586582 | 0.000000000000000 | 0.000000000000000 | 1 | 0.000153008515192 | 0.000000000000000 | 0.000000004473292 | 0.000000000000000 | 0.000000000000000 | 0.000000000000000 | 0.000000000000000 | 0.000000000000000 | 0.000000000000000 | 0.000000000000000 | 0.000000000000000 | 0.000002506136653 | 0.000000336822257 | 0.000000000000000 | 0.000000000000000 | 0.000000000000000 | 0.000000000000000 | 0.000000009876287 | 0.000000093728768 |
| Gut_TS5 | 0.000000087096512 | 0.000000000000000 | 0.000000000000000 | 0.000000000000000 | 0.000000000000000 | 0.000000000000000 | 0.000000461711355 | 0.000346694484037 | 0.000005245421151 | 0.000000000000000 | 0.000000000000000 | 0.000153008515192 | 1 | 0.000012840320157 | 0.000000004101836 | 0.000000000000000 | 0.000000000000000 | 0.000000000000000 | 0.000000000000000 | 0.000000014463349 | 0.000000000000000 | 0.000000000000000 | 0.000000000000000 | 0.000004415797154 | 0.000000492629319 | 0.000000000000000 | 0.000000007817708 | 0.000000000000000 | 0.000000066654702 | 0.000000041153306 | 0.000000000000000 |
| GutlessWorm | 0.000023003912541 | 0.000000000000000 | 0.000000016873641 | 0.000000000000000 | 0.000000000000000 | 0.000016692173386 | 0.000000041582135 | 0.000000011913822 | 0.000000000000000 | 0.000000005049462 | 0.000000006070482 | 0.000000000000000 | 0.000012840320157 | 1 | 0.000007758356138 | 0.000000000000000 | 0.000000000000000 | 0.000000006444865 | 0.000000008120056 | 0.000000054868934 | 0.000018432409205 | 0.000000020760493 | 0.000000000000000 | 0.000000005603942 | 0.000022536369128 | 0.000000024311312 | 0.000000053651226 | 0.000000000000000 | 0.000024268351398 | 0.000019379040886 | 0.000000005680758 |
| HotSpring | 0.000000059468549 | 0.000000001866218 | 0.000000038644286 | 0.000000000000000 | 0.000000000000000 | 0.000000524304972 | 0.000000052255458 | 0.000000040469437 | 0.000000012058630 | 0.000000005895807 | 0.000000343170265 | 0.000000004473292 | 0.000000004101836 | 0.000007758356138 | 1 | 0.000000000000000 | 0.000000000000000 | 0.000000009094010 | 0.000000015599730 | 0.000000133680727 | 0.000000040847050 | 0.000000021352085 | 0.000000000000000 | 0.000000036263313 | 0.000000241382104 | 0.000000019352160 | 0.000000041052329 | 0.000000000000000 | 0.000001782546834 | 0.000000000000000 | 0.000000002291521 |
| KingLIMic | 0.000000000000000 | 0.000021102384509 | 0.000000000000000 | 0.000000000000000 | 0.000000000000000 | 0.000000000000000 | 0.000000000000000 | 0.000000000000000 | 0.000000000000000 | 0.000000000000000 | 0.000000000000000 | 0.000000000000000 | 0.000000000000000 | 0.000000000000000 | 0.000000000000000 | 1 | 0.000162119692610 | 0.000000358423061 | 0.000005890742353 | 0.000001019098849 | 0.000000390151152 | 0.000000000000000 | 0.000000000000000 | 0.000000037576512 | 0.000000229923832 | 0.000000000000000 | 0.000000000000000 | 0.000000000000000 | 0.000000000000000 | 0.000000000000000 | 0.000000289263609 |
| KingLIVir | 0.000000000000000 | 0.000000292066347 | 0.000000083560297 | 0.000000000000000 | 0.000000000000000 | 0.000000000000000 | 0.000000000000000 | 0.000000000000000 | 0.000000000000000 | 0.000000000000000 | 0.000000183919986 | 0.000000000000000 | 0.000000000000000 | 0.000000000000000 | 0.000000000000000 | 0.000162119692610 | 1 | 0.000000000000000 | 0.000000000000000 | 0.000000528258158 | 0.000000000000000 | 0.000000000000000 | 0.000000430936197 | 0.000000000000000 | 0.000000141115195 | 0.000000000000000 | 0.000000043080618 | 0.000000000000000 | 0.000000000000000 | 0.000000000000000 | 0.000001555767080 |
| Madracis | 0.000000031737689 | 0.000000006956063 | 0.000000010439195 | 0.000000000000000 | 0.000001522676306 | 0.000000000000000 | 0.000000000000000 | 0.000000000000000 | 0.000000000000000 | 0.000000000000000 | 0.000000229656339 | 0.000000000000000 | 0.000000000000000 | 0.000000006444865 | 0.000000009094010 | 0.000000358423061 | 0.000000000000000 | 1 | 0.000915218607485 | 0.000000049873707 | 0.000000041700774 | 0.000000000000000 | 0.000000459322063 | 0.000000031105307 | 0.000000073901613 | 0.000000102988860 | 0.000000210370613 | 0.000000258858145 | 0.000000000000000 | 0.000000000000000 | 0.000000116979494 |
| Mussismilia | 0.000000042713457 | 0.000001455633836 | 0.000000027434058 | 0.000000000000000 | 0.000001550757939 | 0.000000000000000 | 0.000000000000000 | 0.000000000000000 | 0.000000000000000 | 0.000000000000000 | 0.000000000000000 | 0.000000000000000 | 0.000000000000000 | 0.000000008120056 | 0.000000015599730 | 0.000005890742353 | 0.000000000000000 | 0.000915218607485 | 1 | 0.000000048324663 | 0.000000307718854 | 0.000000000000000 | 0.000001852711282 | 0.000000013635514 | 0.000000214623102 | 0.000000052032660 | 0.000000064438561 | 0.000000131914170 | 0.000000008355147 | 0.000000000000000 | 0.000000079000784 |
| Polynesia | 0.000000196981166 | 0.000000016082592 | 0.000000000000000 | 0.000000166615260 | 0.000000053316266 | 0.000000108400693 | 0.000000027892978 | 0.000000098920259 | 0.000000030611560 | 0.000000009114601 | 0.000000046524075 | 0.000000000000000 | 0.000000014463349 | 0.000000054868934 | 0.000000133680727 | 0.000001019098849 | 0.000000528258158 | 0.000000049873707 | 0.000000048324663 | 1 | 0.000000119615959 | 0.000000152357780 | 0.000000876943067 | 0.000002284812919 | 0.000003407707183 | 0.000000483736204 | 0.000000538365592 | 0.000000000000000 | 0.000000000000000 | 0.000000000000000 | 0.000003582695567 |
| Porites | 0.000000120329046 | 0.000000207936366 | 0.000000034506606 | 0.000000000000000 | 0.000000000000000 | 0.000000000000000 | 0.000000102409776 | 0.000000000000000 | 0.000000000000000 | 0.000000000000000 | 0.000000000000000 | 0.000000000000000 | 0.000000000000000 | 0.000018432409205 | 0.000000040847050 | 0.000000390151152 | 0.000000000000000 | 0.000000041700774 | 0.000000307718854 | 0.000000119615959 | 1 | 0.000000000000000 | 0.000000000000000 | 0.000000978615843 | 0.000000534530518 | 0.000000127223821 | 0.000000159250089 | 0.000000165054983 | 0.000000292520102 | 0.000000419595110 | 0.000000238788841 |
| RedMine | 0.000000450755807 | 0.000000000000000 | 0.000000047410076 | 0.000000105820431 | 0.000000041930551 | 0.000035812905436 | 0.000000045068856 | 0.000000000000000 | 0.000000052619526 | 0.000000000000000 | 0.000000000000000 | 0.000000000000000 | 0.000000000000000 | 0.000000020760493 | 0.000000021352085 | 0.000000000000000 | 0.000000000000000 | 0.000000000000000 | 0.000000000000000 | 0.000000152357780 | 0.000000000000000 | 1 | 0.000000000000000 | 0.000000000000000 | 0.000000077645413 | 0.000000000000000 | 0.000000000000000 | 0.000000000000000 | 0.000000000000000 | 0.000000016957606 | 0.000000000000000 |
| SARVir | 0.000000000000000 | 0.000000000000000 | 0.000000000000000 | 0.000000000000000 | 0.000000037706302 | 0.000000000000000 | 0.000000000000000 | 0.000000000000000 | 0.000000000000000 | 0.000000000000000 | 0.000000494114566 | 0.000000000000000 | 0.000000000000000 | 0.000000000000000 | 0.000000000000000 | 0.000000000000000 | 0.000000430936197 | 0.000000459322063 | 0.000001852711282 | 0.000000876943067 | 0.000000000000000 | 0.000000000000000 | 1 | 0.000000000000000 | 0.000000000000000 | 0.000000000000000 | 0.000000029772420 | 0.000000212707544 | 0.000000000000000 | 0.000000000000000 | 0.000002319409081 |
| Sludge_M09 | 0.000000021051320 | 0.000000000000000 | 0.000000024099157 | 0.000000000000000 | 0.000000029668888 | 0.000001369336154 | 0.000000109222587 | 0.000000902602193 | 0.000000051973728 | 0.000000012440561 | 0.000000000000000 | 0.000002506136653 | 0.000004415797154 | 0.000000005603942 | 0.000000036263313 | 0.000000037576512 | 0.000000000000000 | 0.000000031105307 | 0.000000013635514 | 0.000002284812919 | 0.000000978615843 | 0.000000000000000 | 0.000000000000000 | 1 | 0.000064490116807 | 0.000000230205880 | 0.000000294314510 | 0.000000000000000 | 0.000000000000000 | 0.000000015257506 | 0.000000037749172 |
| Sludge_V09 | 0.000000191567901 | 0.000000000000000 | 0.000000000000000 | 0.000000241789733 | 0.000000182348458 | 0.000000288856662 | 0.000000000000000 | 0.000000000000000 | 0.000000000000000 | 0.000000015554746 | 0.000000074334168 | 0.000000336822257 | 0.000000492629319 | 0.000022536369128 | 0.000000241382104 | 0.000000229923832 | 0.000000141115195 | 0.000000073901613 | 0.000000214623102 | 0.000003407707183 | 0.000000534530518 | 0.000000077645413 | 0.000000000000000 | 0.000064490116807 | 1 | 0.000000016573215 | 0.000000041166134 | 0.000000000000000 | 0.000000274032882 | 0.000014676226795 | 0.000000000000000 |
| SpongeAb1 | 0.000000160817089 | 0.000000000000000 | 0.000000181278552 | 0.000000000000000 | 0.000000000000000 | 0.000000000000000 | 0.000000000000000 | 0.000000000000000 | 0.000000000000000 | 0.000000000000000 | 0.000000000000000 | 0.000000000000000 | 0.000000000000000 | 0.000000024311312 | 0.000000019352160 | 0.000000000000000 | 0.000000000000000 | 0.000000102988860 | 0.000000052032660 | 0.000000483736204 | 0.000000127223821 | 0.000000000000000 | 0.000000000000000 | 0.000000230205880 | 0.000000016573215 | 1 | 0.000421156964571 | 0.000000059603050 | 0.000000000000000 | 0.000000000000000 | 0.000001142409871 |
| SpongeAb2 | 0.000000512481168 | 0.000000000000000 | 0.000000299110323 | 0.000000000000000 | 0.000000000000000 | 0.000000000000000 | 0.000000000000000 | 0.000000000000000 | 0.000000000000000 | 0.000000009410985 | 0.000000000000000 | 0.000000000000000 | 0.000000007817708 | 0.000000053651226 | 0.000000041052329 | 0.000000000000000 | 0.000000043080618 | 0.000000210370613 | 0.000000064438561 | 0.000000538365592 | 0.000000159250089 | 0.000000000000000 | 0.000000029772420 | 0.000000294314510 | 0.000000041166134 | 0.000421156964571 | 1 | 0.000000000000000 | 0.000000000000000 | 0.000000000000000 | 0.000001746623779 |
| TampaBay | 0.000000000000000 | 0.000000000000000 | 0.000000000000000 | 0.000000000000000 | 0.000000471965009 | 0.000000000000000 | 0.000000000000000 | 0.000000000000000 | 0.000000000000000 | 0.000000000000000 | 0.000007039026661 | 0.000000000000000 | 0.000000000000000 | 0.000000000000000 | 0.000000000000000 | 0.000000000000000 | 0.000000000000000 | 0.000000258858145 | 0.000000131914170 | 0.000000000000000 | 0.000000165054983 | 0.000000000000000 | 0.000000212707544 | 0.000000000000000 | 0.000000000000000 | 0.000000059603050 | 0.000000000000000 | 1 | 0.000000000000000 | 0.000000000000000 | 0.000003341815651 |
| TermiteGut | 0.000000039786245 | 0.000000000000000 | 0.000000000000000 | 0.000000000000000 | 0.000000000000000 | 0.000000052409803 | 0.000000000000000 | 0.000000000000000 | 0.000000000000000 | 0.000000000000000 | 0.000000026236696 | 0.000000000000000 | 0.000000066654702 | 0.000024268351398 | 0.000001782546834 | 0.000000000000000 | 0.000000000000000 | 0.000000000000000 | 0.000000008355147 | 0.000000000000000 | 0.000000292520102 | 0.000000000000000 | 0.000000000000000 | 0.000000000000000 | 0.000000274032882 | 0.000000000000000 | 0.000000000000000 | 0.000000000000000 | 1 | 0.000020533907428 | 0.000000000000000 |
| Waseca | 0.000008767052831 | 0.000000000000000 | 0.000000010239761 | 0.000000058281480 | 0.000000000000000 | 0.000001370441446 | 0.000000011336846 | 0.000000034192446 | 0.000000023522759 | 0.000000004609830 | 0.000010378494289 | 0.000000009876287 | 0.000000041153306 | 0.000019379040886 | 0.000000000000000 | 0.000000000000000 | 0.000000000000000 | 0.000000000000000 | 0.000000000000000 | 0.000000000000000 | 0.000000419595110 | 0.000000016957606 | 0.000000000000000 | 0.000000015257506 | 0.000014676226795 | 0.000000000000000 | 0.000000000000000 | 0.000000000000000 | 0.000020533907428 | 1 | 0.000000000000000 |
| WaterJF1 | 0.000000024412964 | 0.000000005743898 | 0.000000012793536 | 0.000000000000000 | 0.000000000000000 | 0.000000038361261 | 0.000000033747682 | 0.000000102872252 | 0.000000000000000 | 0.000000000000000 | 0.000001095238900 | 0.000000093728768 | 0.000000000000000 | 0.000000005680758 | 0.000000002291521 | 0.000000289263609 | 0.000001555767080 | 0.000000116979494 | 0.000000079000784 | 0.000003582695567 | 0.000000238788841 | 0.000000000000000 | 0.000002319409081 | 0.000000037749172 | 0.000000000000000 | 0.000001142409871 | 0.000001746623779 | 0.000003341815651 | 0.000000000000000 | 0.000000000000000 | 1 |
