# Supplementary material for: Comparative Metagenomic Analysis of Coral Microbial Communities Using a Reference-Independent Approach
Source: PLoS One. 2014 Nov 7;9(11):e111626. doi: 10.1371/journal.pone.0111626 (PMC4224422; doi:10.1371/journal.pone.0111626)
Supplement: Table S3 — Prevalent Pfam domains in the shared sequences of four metagenome pairs. (DOCX) [file pone.0111626.s006.docx]

| **Identical sequences of Gut_TS5 and ChickenCecum** |
| --- |
| PF00593 TonB dependent receptor |
| PF00873 AcrB/AcrD/AcrF family |
| PF07980 SusD family |
| PF07690 Major Facilitator Superfamily |
| PF00005 ABC transporter |
| PF07715 TonB-dependent Receptor Plug Domain |
| PF02321 Outer membrane efflux protein |
| PF04055 Radical SAM superfamily |
| PF00009 Elongation factor Tu GTP binding domain |
| PF02518 Histidine kinase-, DNA gyrase B-, and HSP90-like ATPase |
| **Identical sequences of Mussismilia and Madracis** |
| PF02305 Capsid protein (F protein) |
| PF00910 RNA helicase |
| PF02407 Putative viral replication protein |
| PF01844 HNH endonuclease |
| PF01446 Replication protein |
| PF09295 ChAPs (Chs5p-Arf1p-binding proteins) |
| PF00124 Photosynthetic reaction centre protein |
| PF06280 Fn3-like domain (DUF1034) |
| PF06646 High affinity transport system protein p37 |
| PF01051 Initiator Replication protein |
| **Identical sequences of SpongeAb1 and SpongeAb2** |
| PF00003 7 transmembrane sweet-taste receptor of 3 GCPR |
| PF02811 PHP domain |
| PF01545 Cation efflux family |
| PF06131 Schizosaccharomyces pombe repeat of unknown function (DUF963) |
| PF00023 Ankyrin repeat» found 71 times. |
| PF00530 Scavenger receptor cysteine-rich domain» found 56 times. |
| PF00078 Reverse transcriptase (RNA-dependent DNA polymerase)» found 50 times. |
| PF00102 Protein-tyrosine phosphatase» found 46 times. |
| PF00115 Cytochrome C and Quinol oxidase polypeptide I» found 39 times. |
| PF07714 Protein tyrosine kinase» found 39 times. |
| **Identical sequences of ArcticVIr and GOMVir** |
| PF00501 AMP-binding enzyme |
| PF00593 TonB dependent receptor |
| PF00171 Aldehyde dehydrogenase family |
| PF07715 TonB-dependent Receptor Plug Domain |
| PF00872 Transposase, Mutator family |
| PF07690 Major Facilitator Superfamily |
| PF00106 short chain dehydrogenase |
| PF00848 Ring hydroxylating alpha subunit (catalytic domain) |
| PF00441 Acyl-CoA dehydrogenase, C-terminal domain |
| PF07992 Pyridine nucleotide-disulphide oxidoreductase |
