# Supplementary material for: Comparative Metagenomic Analysis of Coral Microbial Communities Using a Reference-Independent Approach
Source: PLoS One. 2014 Nov 7;9(11):e111626. doi: 10.1371/journal.pone.0111626 (PMC4224422; doi:10.1371/journal.pone.0111626)
Supplement: Table S4 — Node centrality measures. (DOCX) [file pone.0111626.s007.docx]

|  | Betweenness | Clustering Coefficient | Degree |
| --- | --- | --- | --- |
| A.pompejana | 0.02137845 | 0.63684211 | 20 |
| Acropora | 0.00683651 | 0.68181818 | 12 |
| ArcticSoil | 0.01635438 | 0.6 | 15 |
| ArcticVir | 0.00550477 | 0.62222222 | 10 |
| BBCVir | 0.01237566 | 0.6 | 11 |
| BlackMine | 0.01179409 | 0.64761905 | 15 |
| CFLung | 0.01602668 | 0.60952381 | 15 |
| ChickenCecum | 0.00301531 | 0.8 | 11 |
| CowRumen | 0.00187976 | 0.80555556 | 9 |
| ForestSoil | 0.0023285 | 0.75 | 9 |
| GOMVir | 0.0281719 | 0.53333333 | 16 |
| Gut_TS1 | 0.00211866 | 0.77777778 | 9 |
| Gut_TS5 | 0.00784491 | 0.70512821 | 13 |
| GutlessWorm | 0.02515677 | 0.61904762 | 21 |
| HotSpring | 0.04433347 | 0.54940711 | 23 |
| KingLIMic | 0.00186736 | 0.80555556 | 9 |
| KingLIVir | 0.00455483 | 0.58333333 | 9 |
| Madracis | 0.01845123 | 0.66013072 | 18 |
| Mussismilia | 0.02255309 | 0.62745098 | 18 |
| Polynesia | 0.07522746 | 0.49 | 25 |
| Porites | 0.02181597 | 0.64052288 | 18 |
| RedMine | 0.00797567 | 0.63636364 | 12 |
| SARVir | 0.00394149 | 0.72222222 | 9 |
| Sludge_M9 | 0.04278021 | 0.56277056 | 22 |
| Sludge_V09 | 0.05023879 | 0.50649351 | 22 |
| SpongeAb1 | 0.00442907 | 0.85897436 | 13 |
| SpongeAb2 | 0.01460042 | 0.7 | 16 |
| TampaBay | 0.00250784 | 0.71428571 | 8 |
| TermiteGut | 0.00254271 | 0.77777778 | 10 |
| Waseca | 0.02533238 | 0.52205882 | 17 |
| WaterJF1 | 0.04778581 | 0.54285714 | 21 |
